# Preparation of Functional Silica Using a Bioinspired Method

**DOI:** 10.3791/57730

**Published:** 2018-08-01

**Authors:** Joseph R.H. Manning, Eleni Routoula, Siddharth V. Patwardhan

**Affiliations:** ^1^Department of Chemical and Biological Engineering, University of Sheffield

**Keywords:** Chemistry, Issue 138, Porous silica, Encapsulation, nanomaterials, green chemistry, enzyme immobilization

## Abstract

The goal of the protocols described herein is to synthesize bioinspired silica materials, perform enzyme encapsulation therein, and partially or totally purify the same by acid elution. By combining sodium silicate with a polyfunctional bioinspired additive, silica is rapidly formed at ambient conditions upon neutralization.

The effect of neutralization rate and biomolecule addition point on silica yield are investigated, and biomolecule immobilization efficiency is reported for varying addition point. In contrast to other porous silica synthesis methods, it is shown that the mild conditions required for bioinspired silica synthesis are fully compatible with the encapsulation of delicate biomolecules. Additionally, mild conditions are used across all synthesis and modification steps, making bioinspired silica a promising target for the scale-up and commercialization as both a bare material and active support medium.

The synthesis is shown to be highly sensitive to conditions, *i.e.,* the neutralization rate and final synthesis pH, however tight control over these parameters is demonstrated through the use of auto titration methods, leading to high reproducibility in reaction progression pathway and yield.

Therefore, bioinspired silica is an excellent active material support choice, showing versatility towards many current applications, not limited to those demonstrated here, and potency in future applications.

**Figure:**
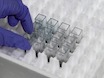


## Introduction

The use of silica as a structural support for industrial catalysts is well established, allowing for the improved catalyst activity, stability and processability,^1^ hence potentially reducing the operating cost. These benefits are compounded in the case of enzyme immobilization, as storage within a silica pore system can confer significant benefits on the enzyme lifetime over its free counterpart. Accordingly, much effort has been expended in finding the best method to attach enzymes to silica species, with multiple reviews comparing investigations using different methods of immobilization on siliceous solid supports.^2^,^3^,^4^

Enzymes are typically attached via physisorption or covalent bonding, in addition to encapsulation within a porous material.^5^ However, there are significant drawbacks related to each method: physisorption relies on transient surface interactions between the silica and biomolecule, which can very easily be weakened by the reaction conditions leading to the unacceptable enzyme leaching. The much stronger covalent attachment usually results in lower activity due to the reduced conformational freedom of the active species. Encapsulation can result in reduced activity due to the enzyme inaccessibility or diffusional limitations.^6^

Recent developments in the field of milder (often dubbed 'bioinspired') silica syntheses have established the *in situ* encapsulation of biomolecules and other active species during the material synthesis.^7^,^8^,^9^ This method negates many of the drawbacks of conventional immobilization - unlike chemisorption approaches the conformational freedom of the biomolecule is maintained by the use of weaker noncovalent interactions but as the pore cavity forms around the biomolecule, leaching is still prevented. Indeed, encapsulation has been demonstrated to work for a range of biomolecules and even whole cells,^10^ and through encapsulation in bioinspired silica effects such as deactivation due to harsh process conditions can be avoided.^7^,^11^

The goal of the method described herein is to prepare a porous silica with controllable properties, under ambient conditions, by using a bioinspired organic additive. The method can be easily modified to include encapsulation of either inorganic or bioorganic molecules, a selection of which shall be shown. We further show a facile method for modifying the as-synthesised materials to achieve desired bulk properties and purification by removing the organic template through acid elution.

Compared to the traditional synthesis of templated porous silica supports (*e.g.,*silica materials templated through supramolecular surfactant assemblies like MCM-41 or SBA-15)^12^ this method is significantly faster and milder, enabling tailored, *in situ* encapsulation without the need for numerous immobilization steps and laborious purification. Furthermore, the use of acid elution rather than calcination opens the possibility of organic surface functionalization.

This method is highly applicable to those working in active species immobilization who have found physisorption or covalent immobilization to be ineffective. It is also useful for those researching process scale-up as the bioinspired synthesis is uniquely positioned for industrialisation compared to conventional templated silica materials.^13^,^14^ This method is not recommended for applications which require an ordered array of pores within the material *e.g.,*for photonics, as the material structure is disordered despite any similarity in bulk properties.

## Protocol

### 1. Preparation of Precursor Solutions (and Optional Encapsulant Solutions)

1. Into a 180 mL plastic container, measure 1.5 mmol of sodium silicate pentahydrate (318.2 mg), and dissolve in 20 mL of deionized water.
2. Similarly, in a second container, measure 0.25 mmol of pentaethylene hexamine (PEHA, 58.1 mg) and dissolve in 20 mL of deionized water.
  1. When using alternative amine-containing compounds *e.g.,*diethylenetriamine (DETA) or triethylenetetraamine (TETA), ensure that the total Si:N mole ratio remains constant at 1 (*i.e.,* corresponding to 0.5 mmol of DETA or 0.375 mmol of TETA in the described procedure)^15^.
  2. When using polymeric amine additives *e.g.,*poly(ethyleneimine) (PEI) or poly(allylamine hydrochloride) (PAH), maintain a concentration of 1 mg/mL (final reaction volume)^15^. CAUTION: Handle these amines only inside a fume hood, as they are corrosive or toxic in their pure forms (especially as vapors).
3. To perform *in situ* encapsulation during synthesis, dissolve a pre-determined mass of protein (herein 50 mg of Bovine Serum Albumin, BSA) in 5 mL of deionized water. Subtract this amount of water from the volume of deionized water to be used for the dissolution of sodium silicate pentahydrate.
  1. To ease the protein dissolution without altering its structure, once mixed with deionized water, cap the container and store at 4 °C. Check occasionally on the dissolution progress, preferably without stirring.

### 2. Silica Synthesis

1. Combine the solutions of sodium silicate pentahydrate and PEHA in one of the 180 mL container and add sufficient deionized water to make the final solution volume 41 mL (or 46 if *in situ* encapsulation is omitted).
2. Place the freshly-prepared mixture of sodium silicate and PEHA solutions on the top of a stirrer plate, adding a stirrer bar to provide consistent mixing.
3. Into this vessel, suspend a pH probe and record the initial pH.
  1. At this stage, optionally, remove a 750 µL aliquot of the starting mixture for later determination of the initial [Si] concentration using the molybdenum blue spectrophotometric assay, as described in step 8.1.
4. Begin the synthesis by adding a predetermined quantity of 1 M HCl, as calculated from Figure 1, and observe the immediate evolution of turbidity (see Figure 2)
5. As soon as the acid addition is over, add the encapsulant solution (if any) as quickly as possible. NOTE: The final volume given these quantities is 50 mL of total reaction mixture, leading to Si and N concentrations of 30mM. This can be scaled as desired by multiplying all above quantities by a constant amount.
6. Record the pH after 5 min to determine the reaction completion; ensure that the pH is 7 ± 0.05.

### 3. Acid Elution of the Materials

1. Modify the composition of produced silica after the reaction has reached completion (either as an as-made coagulum or by resuspending a previous synthesized sample of silica) by the addition of further acid.
2. If resuspending silica, mix approximately 150 mg as-prepared bioinspired silica with 100 mL of deionized water in a 180 mL plastic container, and place on the top of a stirrer plate.
3. Once the suspension is well mixed, suspend a pH probe in the vessel.
4. Titrate in further HCl until the desired pH (between 7 and 2) has been reached and allow to stabilize for ca. 1 min.
5. Wait a further 5 min to ensure the system has fully equilibrated, and then proceed to isolate the solid silica.

### 4. Silica Separation and Drying

1. Decant the bioinspired silica suspension into 50 mL centrifuge tubes.
2. Centrifuge the suspension at 5,000 g for 15 min.
3. Remove the supernatant after centrifugation and store for further analysis (*e.g., *Bradford assay, see below). Refill the centrifuge tubes with deionized water, and re-suspend the silica using a vortex mixer.
4. Repeat the centrifugation, supernatant storage, and re-suspension twice.
5. After the final centrifugation, remove the supernatant and scrape the silica into a ceramic crucible.
6. Dry in an oven overnight at 85 °C.
  1. If encapsulation has taken place, use a freeze-drying facility or an oven operating under vacuum to avoid protein denaturation.

### 5. Production of Molybdenum Blue Reagent (MBR) for [Si] Determination

1. To a plastic 1 L volumetric flask, add 8 mmol (10 g) ammonium molybdate tetrahydrate in a fume cupboard.
2. Dissolve this in 500 mL deionized water under stirring.
3. Acidify the solution by carefully adding 60 mL of 10 M HCl solution.
4. Adjust the final volume to 1 L.

### 6. Production of para-aminophenol sulfate reducing agent (RA) for [Si] determination

1. Place a 500 mL glass volumetric flask in a water bath at ambient temperature on a stirrer plate in a fume- cupboard.
2. Add 111 mmol (10 g) of anhydrous oxalic acid, 19.5 mmol (3.35 g) of para-aminophenol sulfate, and 16 mmol (2 g) of sodium sulfite, and dissolve in 250 mL water.
3. Carefully and slowly add 92 g (50 mL) of saturated sulfuric acid while stirring and wait for the solution to cool.
4. Finally, dilute to 500 mL with deionized water.

### 7. Silicomolybdic Acid Assay on Monomeric Silica Species

1. In a 5 mL plastic vial, dilute 300 µL of MBR produced in step 5.4 with 3 mL of deionized water.
2. Add 10 µL of a silicic acid test solution and shake to mix. NOTE: This solution will slowly turn yellow.
3. After exactly 15 min, add 1.6 mL of the reducing agent prepared from section 6 to reduce the yellow silicomolybdate complex to its blue isomer.
4. Allow a blue color to develop for at least 2, but not more than 24 h.
5. Measure sample absorbance at 810 nm in a UV-vis spectrophotometer and calculate [Si] against a calibration curve.

### 8. Silico Molybdic Acid Assay on Polymeric Silica Species

1. To measure the concentration of polysilicate species using the molybdenum blue method, in a microcentrifuge tube, combine 750 µL of 2 M sodium hydroxide solution with 750 µL silica suspension.
2. Seal and place in a microcentrifuge float.
  1. Ensure sufficient headspace is left in the tube to prevent bursting due to pressure build up. NOTE: A headspace of 500 µL is usually sufficient to avoid this. Alternatively, the procedure can be carried out in open vials so long as liquid loss due to evaporation is accounted for.
3. Float the microcentrifuge tubes in a water bath heated to 80 °C and leave it to dissolve for 1 h.
4. After 1 h has elapsed, remove the microcentrifuge tubes and wipe the outside dry.
5. Once cooled, [Si] can be determined as described above as described in steps 7.2 to 7.5.

### 9. Bradford Assay Procedure for Determination of Protein Concentration in Silica

1. Insert a predetermined amount of (room temperature) Bradford reagent and sample in each assigned cuvette (see **Table 1** and **Table 2** for specific volumes). Use disposable pipette tips for every cuvette to avoid volume alterations due to the nature of the reagent and repeat each point in triplicate.
2. Mix each cuvette by inverting 3 times and leave to develop for 10 min.
3. Measure absorbance at 595 nm using pure supernatant as blank.
4. Calculate the original absorbance of each cuvette by subtracting from each measurement the absorbance found for the control sample (cuvette No. 0 in both assays).
5. Calculate the protein concentration of Unknown sample using a calibration curve (Figure 3). In case of dilution of the original sample, the dilution factor needs to be accounted for.
  1. Create a calibration curve for each set of experiments by plotting measured absorbance against concentration of BSA to avoid random fluctuations that might affect the assay's sensitivity.
  2. Although this protein assay is meant to use BSA as a standard to quantify any type of protein, create a calibration curve for each specific protein of interest for improved accuracy.
  3. If the protein content of the unknown sample is expected to be higher than the covered range of the calibration curve, dilute it as needed.
6. Determine protein content for each sample during re-suspension to monitor possible protein loss.

## Representative Results

The techniques described above are able to consistently and reproducibly precipitate silica. This is easiest to determine by the rapid onset of turbidity within the reaction vessel, which upon cessation of agitation will spontaneously settle into a thick coagulum of precipitated silica (Figure 2). The extent of reaction and hence yield can be confirmed by measuring the mass of this coagulum after separation and is typically 58 ± 6.5% (Figure 4, yellow).

Further insight into the reaction progression can be generated by adapting the molybdenum blue spectroscopic method to detect the amount of unreacted monomeric silicate species as well as those species which have reacted to form polysilicates or 'oligomers', but have not managed to reach sufficient size to coagulate (Figure 4, red and blue respectively).

This specific silica speciation data is of particular interest when comparing different titration efficiencies for the precipitation reaction - I.E. how the final reaction pH and the rate at which this is reached affects the polymerization of monomeric silica to an 'oligomer' and its subsequent coagulation to solid silica. By modifying the amount of acid added in stage 2.4 slightly, under- or over-titration of the reaction mixture can be performed (Figure 5). By measuring the silica speciation again for these two cases, a clear difference can be seen in the reaction completion (Figure 4) despite only minor changes to the titration profile of the reaction (Figure 5).

Although no difference is present between the consumption of monomeric species for the three reaction cases (remaining between 29 - 33%), there is a clear difference in the amount of oligomeric silica species which precipitate in each case. This is in agreement with traditional theory on sol-gel silicas - in the 'undershoot' case the pH is held higher for longer, allowing for individual particles to grow and hence aiding efficient coagulation; in the 'overshoot' case the coagulation is induced much faster due to the rapid titration, hence fewer of the silica species have grown to a sufficient size to coagulate and remain trapped in the colloid phase.^16^

Given the importance of titration upon silica formation, *a priori* knowledge of the appropriate titration volume is essential. Although not extractable from the reaction stoichiometry due to the complex protonation behavior of the amine additives and change in silica surface acidity on coagulation, highly reliable empirical relationships between system contents, concentrations and titer volumes are readily generated (Figure 1).

Once coagulation has been completed, material surfaces can be readily modified through the use of acid elution, as has recently been reported by the authors elsewhere.^13^ This allows for fine-tuning of material properties such as composition, porosity, and chemical activity of additive (Figure 6a **and b**).

In this study, BSA was used as an exemplar encapsulant enzyme, however, the techniques described here can be used for multiple enzymes^17^,^18^. The procedure followed for protein detection is the Bradford assay protocol,^19^ using the supernatants stored from each centrifugation cycle. The amount of protein in the supernatant is calculated using a calibration curve created from known amounts of BSA dissolved in the supernatant of a sample with zero protein content (Control sample). The amount of protein encapsulated into silica will be calculated by subtraction of the detected protein in supernatants from the initial amount of protein added. The only reagent needed for the assay is the Bradford Reagent (either procured or made according to standard recipes).

There are three types of assay format, depending on the sample volume, the expected amount of protein to be detected and the measurement method used. Herein, the followed format is specified for a spectrophotometer, requires disposable cuvettes of macro and of micro size and can detect from 10 µg/mL to 1.4 mg/mL of protein.

In Figure 7 the amount of protein detected after each wash (step 4.3) is shown as a % of the initial protein amount (which was 50 mg). Around ~50% of BSA was detected in the supernatant after the first centrifugation, which relates to ~50% immobilization efficiency. As there was no BSA detected in the following washes, BSA (or any other enzyme) could be securely encapsulated during silica synthesis with no leaching - this is a significant advantage of this method. In order to confirm the presence of BSA in the silica produced, Fourier Transform Infrared Spectroscopy (FTIR) analysis was performed. The presence of the characteristic bands of amide I and II in the area of 1,500/cm and 1650/cm (Figure 8) in the samples prepared in the presence of BSA, but not in the control samples (no BSA) confirmed the presence of BSA in the solids.

In addition to the method of enzyme addition described above (BSA added during neutralization of reaction mixture), there are other possible variations *e.g.,*BSA addition during mixing of the silicate and the additive solutions, prior to neutralization or enzyme added to the silicate or additive solution before their mixing and neutralization. Some of these possibilities were explored further and the immobilization efficiencies (mass of BSA immobilized as a percentage of enzyme added to the reaction system, calculated based on the Bradford assay) and the amount of BSA in the final silica were measured (concentration of BSA in silica as a percentage of the total composite weight produced, see Figure 9). It was clear that when BSA was added to the unreacted reagents (cases A-C in Figure 9) there were no considerable differences in the immobilization efficiency or the amount of BSA in the resulting composite. However, when BSA is added during silica formation (case D in Figure 9), immobilization efficiency and the amount of BSA in the final product were both significantly lower. Despite these differences, the average amount of silica produced remained unchanged (between 85-90 mg). These observations can be explained on the basis of the ionization (or isoelectric point) of BSA, silicate/silica and the additive. The different methods of addition allow for different interactions between the enzyme and silica precursors. As the pH at the time of the addition of the enzyme changes, the ionization of each species will determine intermolecular interactions, which in turn will control the immobilization efficiency.

**Table:** 

**Cuvette No**	**Concentration of BSA (mg/mL)**	**Bradford reagent (mL)**	**Sample (mL)**
0	0 (control)	1.5	0.05
1	0.1	1.5	0.05
2	0.25	1.5	0.05
3	0.5	1.5	0.05
4	0.75	1.5	0.05
5	1	1.5	0.05
6	1.25	1.5	0.05
7	Unknown sample (X)	1.5	0.05

**Table 1: Macro Bradford assay set-up and calculated component volumes.** Valid for determination range 0.1-1.4mg/mL (volumes for 1 replicate)

**Table:** 

**Cuvette No**	**Concentration of BSA (ug/mL)**	**Bradford reagent (mL)**	**Sample (mL)**
0	0 (control)	1	1
1	1	1	1
2	2.5	1	1
3	5	1	1
4	7.5	1	1
5	10	1	1
6	Unknown sample (X)	1	1

**Table 2**: **Micro Bradford assay set-up and calculated component volumes.** Valid for determination range 1-10 µg/mL (volumes for 1 replicate)

**Figure:**
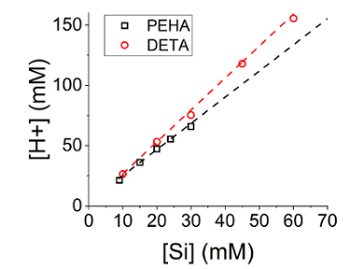

Figure 1**:** **Required titer volume against silica concentration for reaction systems using either DETA or PEHA as the additive**. Silica was synthesized at varying concentrations while maintaining an [N]:[Si] ratio of 1, for two different additive chemicals. Error bars are one standard deviation around the mean.

**Figure:**
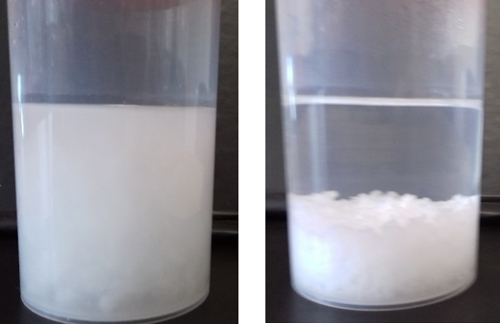

Figure 2**:** **Photographs of silica coagulum in the reaction vessel (a) during and (b) after agitation, demonstrating the solution turbidity and settling that are indicative of an optimal reaction.** 

**Figure:**
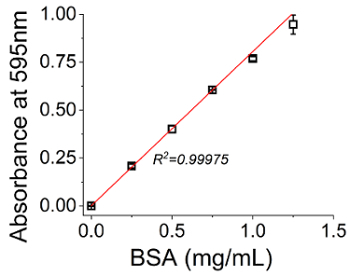

Figure 3**: Exemplar calibration curve for Bradford macro assay.** Supernatant from bioinspired silica synthesis in absence of BSA is mixed with a known amount of the protein, after which Bradford analysis is performed as described in step 9.1.

**Figure:**
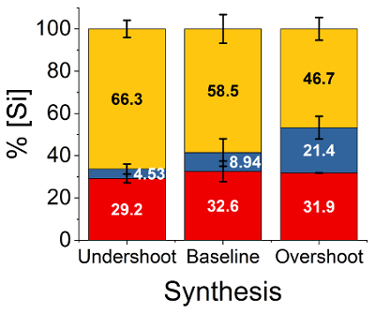

Figure 4**: Final polymerization states of silica species for different reaction conditions.** Silica is synthesized using optimal (baseline) conditions, as well as with over- or under-titration, after which relative silica concentration is quantified for monomeric or dimeric silicates (red), polysilicate 'oligomers' (blue) and unstable coagulating silica (yellow).

**Figure:**
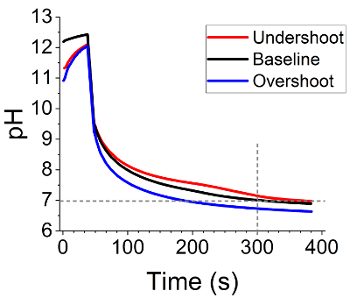

Figure 5**:Progression of pH through reaction system as a function of initial titer volume.** Acid is immediately dosed after ca. 38s of mixing, causing the pH to rapidly drop to below 8. Afterward, further quantities of acid are automatically dosed such that the pH was 7.0 ± 0.05 300s after initial addition. In the case of over-titrating, this was not achievable, as the initial dose was sufficient to drop the pH below 7, reaching pH 6.65 after 300s. Initial HCl volume added for 'undershoot,' 'baseline,' and 'overshoot' was 6.90, 7.05, and 7.20mL respectively. 

**Figure:**
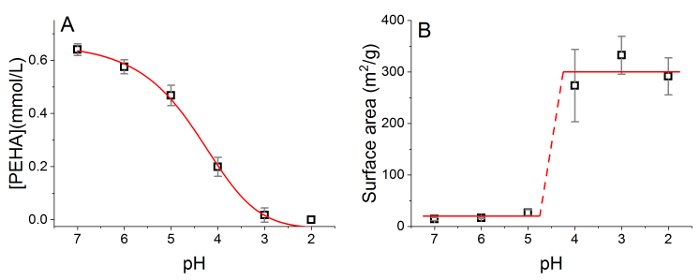

Figure 6**:** **Representative property changes upon acidification of coagulated silica material.** (a) Change of additive concentration with respect to pH, and (b) change of silica porosity with respect to pH. Reproduced from Manning* et al. *13 under Creative Commons license. 

**Figure:**
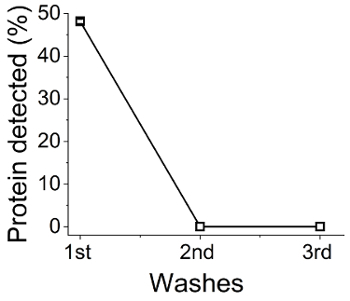

Figure 7**: BSA concentration in bioinspired silica synthesis supernatants**. Bradford assays were carried out on reaction supernatants after centrifugation, from which the relative amount remaining (therefore occluded from the synthesized silica) was determined. 

**Figure:**
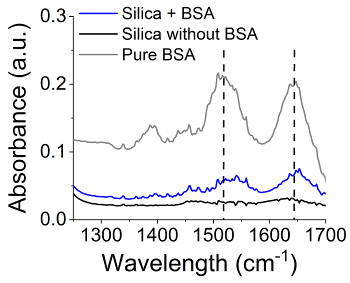

Figure 8**:** **FTIR analysis on bioinspired silica with and without active species encapsulation**. Spectra showed: black line: bioinspired silica, gray line: pure BSA, blue line: bioinspired silica loaded with BSA. Vertical dashed lines indicate characteristic amide bands. 

**Figure:**
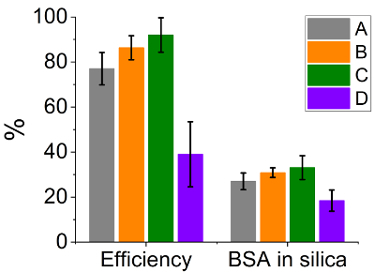

Figure 9**:Immobilization efficiency and the amount of BSA in the composite for silica produced using PEHA.** BSA was added **(A)** in the PEHA solution before mixing with silicate, (**B)** in the silicate solution before mixing with PEHA, **(C)** after initial mixing of PEHA and silicate solutions, and **(D) **after mixing PEHA and silicate solutions and neutralizing. Efficiency is measured as% BSA encapsulated from the reaction mixture as a proportion of total BSA added, while BSA in silica signifies% concentration of BSA in final silica composite by mass. Error bars are one standard deviation around the mean.

## Discussion

In the current work, we present a method for rapidly precipitating bioinspired silica materials and encapsulation of biomolecules therein. We demonstrate critical steps within the procedure, namely the amount of reaction-initiating acid to be added, and timing of addition of the biomolecule encapsulant. We show the effect of acid addition amount on both reaction progression and yield (Figure 4 and Figure 5, respectively), and demonstrated a method for tight control over synthesis conditions, allowing for consistency despite this sensitivity. Regarding active species encapsulation, although straightforward in terms of procedure, encapsulation is shown to be sensitive to the conditions of the experiment (order of addition, pH of addition, environmental conditions), however, consistency in material properties is again achievable.

The synthesis conditions can be modified through the use of different additives, many of which have been published elsewhere,^15^ providing a range of morphologies and porosities. Further, post-synthetic techniques to modify and chemically tailor bioinspired silica materials have been reported such as mild purification^13^ and surface amine decoration.^20^ Finally, due to the mild, aqueous nature of the synthesis, *in situ* encapsulation is possible for a wider range of substrates than those demonstrated here, ranging from enzymes^17^,^18^ to whole cells,^21^ metal salts,^22^ active pharmaceutical ingredients,^23^ and quantum dots.^24^

Unlike other organic-mediated silica syntheses (such as the MCM-41 or SBA-15 family of materials), the polyfunctional nature of bioinspired additives cannot produce ordered pore structures, nor highly monodisperse particle-size distributions characteristic of Stöber-type silica.^25^ This is due to the lack of well-defined micellization behavior of bioinspired additives (outside of special cases)^26^ coupled with their increased catalytic activity over monofunctional amine-containing additives.^26^

On the other hand, this polyfunctional additive nature enables the use of shorter reaction times and milder temperature & pressure compared to other organic-mediated silica syntheses. This also leads to the possibility of room-temperature additive elution as described above, which has yet to be achieved for these other silica families due to the specifics of their surface chemistry.^27^,^28^,^29^ Consequently, bioinspired silica materials have been shown to be both more economical and practical to produce at a larger scale, leading to easier commercialization and development.^14^

In summary, bioinspired silica synthesis represents a rapid, facile method for producing active species supports or gas sorbent media. Through tight control of pH during and after the reaction, a wide array of silica-amine composites can be synthesized with varying properties, which is further complemented by the possibility of *in situ* encapsulation of an array of different organic, inorganic, or bio-organic materials. Although independent post-synthetic modification of bioinspired additive and encapsulant concentration has yet to be achieved, these methods represent a promising step towards environmentally benign chemical processes.

## Disclosures

The authors declare no competing financial interest.

## References

[B0] Swaisgood HE (2004). The use of immobilized enzymes to improve functionality. Proteins Food Process.

[B1] Hartmann M, Kostrov X (2013). Immobilization of enzymes on porous silicas - benefits and challenges. Chem Soc Rev.

[B2] Hudson S, Cooney J, Magner E (2008). Proteins in Mesoporous Silicates. Angew Chemie Int Ed.

[B3] Hanefeld U, Gardossi L, Magner E (2009). Understanding enzyme immobilisation. Chem Soc Rev.

[B4] Magner E (2013). Immobilisation of enzymes on mesoporous silicate materials. Chem Soc Rev.

[B5] Rodrigues RC, Ortiz C, Berenguer-Murcia Á, Torres R, Fernández-Lafuente R (2013). Modifying enzyme activity and selectivity by immobilization. Chem Soc Rev.

[B6] Forsyth C, Patwardhan SV (2014). Bio-Inspired Silicon-Based Materials.

[B7] Luckarift HR, Spain JC, Naik RR, Stone MO (2004). Enzyme immobilization in a biomimetic silica support. Nat Biotechnol.

[B8] Betancor L, Luckarift HR (2008). Bioinspired enzyme encapsulation for biocatalysis. Trends Biotechnol.

[B9] Livage J, Coradin T, Roux C (2001). Encapsulation of biomolecules in silica gels. J Phys Condens Matter.

[B10] Hartmann M, Jung D (2010). Biocatalysis with enzymes immobilized on mesoporous hosts: the status quo and future trends. J Mater Chem.

[B11] Carlsson N, Gustafsson H, Thörn C, Olsson L, Holmberg K, Åkerman B (2014). Enzymes immobilized in mesoporous silica: A physical-chemical perspective. Adv Colloid Interface Sci.

[B12] Manning JRH, Yip TWS, Centi A, Jorge M, Patwardhan SV (2017). An Eco-Friendly, Tunable and Scalable Method for Producing Porous Functional Nanomaterials Designed Using Molecular Interactions. ChemSusChem.

[B13] Drummond C, McCann R, Patwardhan SV (2014). A feasibility study of the biologically inspired green manufacturing of precipitated silica. Chem Eng J.

[B14] Patwardhan SV (2011). Biomimetic and bioinspired silica: recent developments and applications. Chem Commun.

[B15] Iler RK (1979). The Chemistry of Silica: Solubility, Polymerization, Colloid and Surface Properties and Biochemistry of Silica.

[B16] Forsyth C, Yip TWS, Patwardhan SV (2013). CO2 sequestration by enzyme immobilized onto bioinspired silica. Chem Commun (Camb).

[B17] Forsyth C, Patwardhan SV (2013). Controlling performance of lipase immobilised on bioinspired silica. J Mater Chem B.

[B18] Bradford MM (1976). A rapid and sensitive method for the quantitation of microgram quantities of protein utilizing the principle of protein-dye binding. Anal Biochem.

[B19] Ewlad-Ahmed AM, Morris MA, Patwardhan SV, Gibson LT (2012). Removal of formaldehyde from air using functionalized silica supports. Environ Sci Technol.

[B20] Yang SH, Ko EH, Jung YH, Choi IS (2011). Bioinspired functionalization of silica-encapsulated yeast cells. Angew Chemie.

[B21] Alotaibi KM (2017). Iron supported on bioinspired green silica for water remediation. Chem Sci.

[B22] Davidson S, Lamprou DA, Urquhart AJ, Grant MH, Patwardhan SV (2016). Bioinspired Silica Offers a Novel, Green, and Biocompatible Alternative to Traditional Drug Delivery Systems. ACS Biomater Sci Eng.

[B23] Patwardhan SV, Perry CC (2010). Synthesis of enzyme and quantum dot in silica by combining continuous flow and bioinspired routes. Silicon.

[B24] Nozawa K (2005). Smart control of monodisperse stöber silica particles: Effect of reactant addition rate on growth process. Langmuir.

[B25] Belton DJ, Patwardhan SV, Annenkov VV, Danilovtseva EN, Perry CC (2008). From biosilicification to tailored materials: optimizing hydrophobic domains and resistance to protonation of polyamines. Proc Natl Acad Sci U S A.

[B26] de Ávila SG, Silva LCC, Matos JR (2016). Optimisation of SBA-15 properties using Soxhlet solvent extraction for template removal. Microporous Mesoporous Mater.

[B27] Cassiers K, Van Der Voort P, Vansant EF (2000). Synthesis of stable and directly usable hexagonal mesoporous silica by efficient amine extraction in acidified water. Chem Commun.

[B28] Tanev PT, Pinnavaia TJ (1996). Mesoporous Silica Molecular Sieves Prepared by Ionic and Neutral Surfactant Templating: A Comparison of Physical Properties. Chem Mater.

